# Selective Sorption of Cerium Ions from Uranium-Containing Solutions by Remotely Activated Ion Exchangers

**DOI:** 10.3390/polym15040816

**Published:** 2023-02-06

**Authors:** Talkybek Jumadilov, Ainamgul Utesheva, Juozas Grazulevicius, Aldan Imangazy

**Affiliations:** 1 Laboratory of Synthesis and Physicochemistry of Polymers, Bekturov Institute of Chemical Sciences, 106 Sh. Ualikhanov Street, Almaty 050010, Kazakhstan; 2 School of Chemical Engineering, Kazakh-British Technical University, 59 Tole bi Street, Almaty 050000, Kazakhstan; 3 Department of Polymer Chemistry and Technology, Kaunas University of Technology, 73 K. Donelaičio Street, 44249 Kaunas, Lithuania

**Keywords:** ion exchangers, interpolymer system, remote interaction, uranium ions, cerium ions, sorption

## Abstract

This study investigated the effect of the remote activation of the ion exchangers Amberlite IR120 (H^+^ form) and AV-17-8 (OH^−^ form) in aqueous media to increase the sorption activity of the interpolymer system “Amberlite IR120H:AV-17-8” (X:Y, molar ratio of ionic groups) towards cerium ions from uranium-containing solutions. The sorption properties of the above-mentioned interpolymer system with molar ratios X:Y of 6:0, 5:1, 4:2, 3:3, 2:4, 1:5, and 0:6 were studied using the methods of conductometry, gravimetry, and inductively coupled plasma–optical emission spectrometry. The presented research revealed the dependence of the sorption activity of the interpolymer system “Amberlite IR120H:AV-17-8” (X:Y) on the acidity of the solution. At pH 2.0, the highest cerium ion sorption degree from the model solution (containing both cerium and uranium ions) by the interpolymer system “Amberlite IR120H:AV-17-8” (4:2) was 56% after 48 h of interaction, whereas the cerium ion sorption degrees by raw Amberlite IR120H (6:0) and raw AV-17-8 (0:6) were 30% and 0%, respectively. The increased sorption ability of the interpolymer system “Amberlite IR120H:AV-17-8” (4:2) might be associated with the achievement of the highest ionization degree by this system remotely activated in an aqueous medium. Moreover, the cerium ion desorption study demonstrated a 60% degree of desorption using 2M nitric acid as a desorbing agent (eluent). The obtained results demonstrate the potential of using the remote interaction effect for the activation of the ion exchangers in aqueous media as an interpolymer system for increased cerium ion sorption from uranium-containing solutions.

## 1. Introduction

Today, rare earth metals (REMs) are widely used in many industries, causing an increased demand for their production [1,2,3]. However, the manufacturing of high-purity rare-earth elements is a challenging task, since REM-containing ores might contain such radioactive elements as uranium, thorium, and their half-life products. In order to reduce the mining costs for valuable elements, it is urgent to improve traditional approaches or search for new technological methods for obtaining individual elements with a high purity [4,5,6]. Cerium, as the representative of the lanthanide family, belongs to the light REM group and is the most abundant REM in the upper earth crust. In a highly pure state, cerium is a malleable metal with a bright silvery color that rapidly oxidizes in air [7]. Cerium has wide-ranging applications as an alloying addition for significantly improving metal products [8]. Its alloys with magnesium and aluminum have increased strength. For instance, the addition of only 1% cerium to magnesium significantly increases its tensile strength. In addition, to increase the heat resistance and durability of chromium and nickel alloys, cerium is added as an alloying additive [9]. Moreover, pure cerium is a good absorber of most gases by chemisorption. This rare earth metal is also widely used for obtaining pyrotechnic compositions, as its powder is pyrophoric (self-igniting when in contact with air). Additionally, cerium compounds are widely used as catalysts in petrochemical production [10,11]. The demand for cerium is growing annually, but its availability on Earth is limited [12]. However, most uranium mining enterprises generate cerium in their process fluids as a co-product, and its recovery could provide an additional supply of this rare earth element [13,14].

Nowadays, there are two main methods for the recovery of cerium from solutions, sorption [15] and liquid–liquid extraction [16]. Of these, sorption methods have recently gained the lead, since they are more environmentally friendly and involve fewer technological cycles [17]. These adsorption techniques use diverse materials, such as biological materials, activated carbons, and activated polymers, which have recently aroused increased interest in the recovery of various metals from aqueous media [18,19,20,21,22,23].

A preferred method for cerium ion recovery from uranium-containing solutions may be the use of commercially available and preliminary activated ion exchangers—for example, the combination of Amberlite IR120 and AV-17-8 as the interpolymer system “Amberlite IR120:AV-17-8 (X:Y, molar ratio of ionic groups) [24,25,26]. The use of an interpolymer system allows the sorption of cerium ions with enhanced efficiency [27]. The goal of this research was to study the selective sorption of the remotely activated ion exchangers in the interpolymer system “Amberlite IR120:AV-17-8” (X:Y, molar ratio of ionic groups) for cerium ion recovery from uranium-containing solutions. The novelty of this study lies in the fact that for the first time, an interpolymer system of activated ion exchangers was used for the selective sorption of cerium ions from a solution containing a radioactive element as a contaminant.

## 2. Materials and Methods

### 2.1. Equipment

For this research, the following materials were used: (1) strongly acidic Amberlite IR120 H (H^+^ form) (Lenntech, Delfgauw, The Netherlands) gel-type cation exchanger based on sulfonated styrene and divinylbenzene copolymer (with granule size 0.50–0.75 mm); and (2) strongly basic AV-17-8 (OH^−^ form) (Azot, Cherkassy, Ukraine) gel-type anion exchanger based on a copolymer of styrene and divinylbenzene (with granule size 0.3–1.2 mm).

In addition, the following reagents were used: cerium (III) nitrate hexahydrate (99.999% trace metal basis, Sigma-Aldrich, Darmstadt, Germany) for the preparation of a cerium-containing solution with a concentration of 6.03 mg/L; and commercial desorbate (a solution with a uranium content of 76.63 g/L) obtained by the “National Atomic Company Kazatomprom” JSC (Astana, Kazakhstan) enterprise during the desorption of uranium from ionites used in the process. For the experiments, the desorbate was diluted to a concentration of 6.03 mg/L. To study the sorption activity of the interpolymer system, previously prepared solutions of cerium and uranium were mixed to obtain a common solution; sulfuric acid (H_2_SO_4_) was used for pH regulation; and diluted nitric acid (HNO_3_) was applied as a desorbing agent (eluent).

For the mass determination of the ion exchangers, a Sartorius Cubis MSE125P-100-DO electronic analytical balance (Sartorius AG, Göttingen, Germany) was used. For the measurement of the hydrogen ion concentrations in the solutions, a “Metrohm 827 pH–Lab” pH meter (Metrohm AG, Herisau, Switzerland) was applied. An iCAP 7400 Duo inductively coupled plasma–optical emission spectrometer (Thermo Fisher Scientific, Winsford, UK) with a wavelength range of 166 to 847 nm was used for the analysis and quantitation of cerium in both liquid and polymer samples. The measurement error was less than 1%.

### 2.2. Determination of the Polymer Chain Binding Degree

The polymer chain binding degree (θ) specifies the ratio of the amount of cerium ions adsorbed to the total amount of ionic groups in the Amberlite IR120H and AV-17-8 ion exchangers. This ratio was used to measure the efficiency of the use of functional groups from the adsorbents side, which strongly depends on the ratio of the amount of ions added to the functional groups and the equilibrium. It was calculated according to Equation (1):

(1) $$\theta =\frac{\vartheta_{\mathrm{sorbed}}}{\vartheta_{\mathrm{is}}}\times 100\%$$

where ϑ_sorbed_ is the amount of sorbed cerium ions (in mol), and ϑ_is_ is the amount (in mol) of ionic groups in the interpolymer system “Amberlite IR120:AV-17-8” (X:Y).

### 2.3. Preparation of the Interpolymer System

For the preparation of the interpolymer system “Amberlite IR120:AV-17-8” (X:Y, molar ratio of ionic groups), we used (1) strongly acidic Amberlite IR120 (hydrogen form) and (2) strongly basic AV-17-8 (hydroxide form). For clarification, an interpolymer system comprises a pair of cross-linked polymers with active functional groups that are present in an aqueous solution but without mutual contact (remote interaction) [28].

### 2.4. Activation of the Interpolymer System

**Figure 1:**
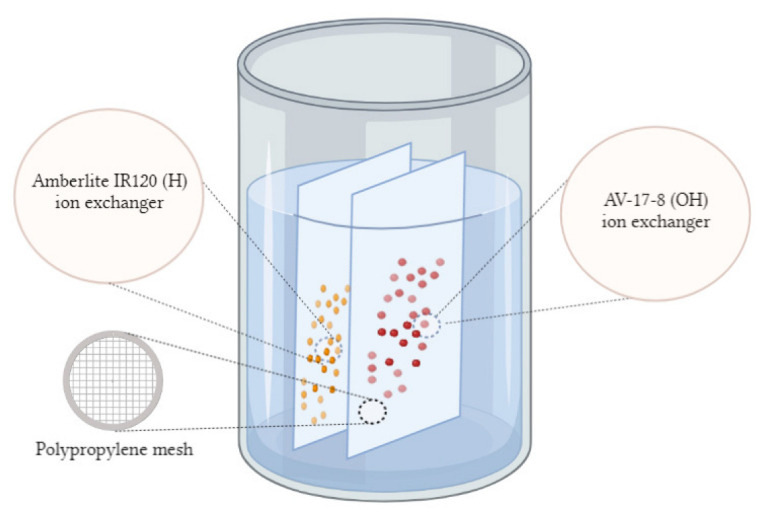
Illustration of the activation process of the interpolymer system “Amberlite IR120:AV-17-8” (X:Y) by remote interaction in the aqueous solution.

Previous studies [29,30,31] showed that the mutual activation of polyelectrolytes during their remote interaction promotes the transition of polymers to a highly ionized state, followed by a significant increase in the sorption degree of target metal ions compared with the raw (unactivated) ion exchangers. For activation, polypropylene meshes containing swollen ion exchangers (Figure 1) were placed opposite each other in a glass with distilled water at a distance of about 1–2 cm, representing the interpolymer system “Amberlite IR120:AV-17-8” (X:Y) (Figure 1).

The acid–base properties of ion exchangers were associated with the dissociation of Amberlite IR120 (H^+^ form) and AV-17-8 (OH^−^ form) with the formation of free H^+^ and OH^−^ ions, respectively [32]. Then, the released H^+^ and OH^−^ ions formed weakly dissociated water molecules and left the functional groups of polyelectrolytes activated and stabilized by intramolecular interactions. As a result, the concentrations of oxonium (H_3_O^+^) and OH^−^ ions became significantly higher around the Amberlite IR120 and AV-17-8, respectively. This gradient presumably made the concentrations of neutral water around the ion exchangers in this interpolymer system lower than the concentrations around the ion exchangers used individually and enhanced the dissociation of counter ions from the ionic groups in the polyelectrolytes.

### 2.5. Determination of the Cerium Ion Concentration and Desorption Studies

For the experiments, the solution containing cerium and the diluted uranium desorbate were mixed to obtain a model solution (1000 mL) with cerium and uranium concentrations (6 mg/L) in order to study the sorption activity of the interpolymer system “Amberlite IR120:AV-17-8” (X:Y) with molar ratios of 6:0, 5:1, 4:2, 3:3, 2:4, 1:5, and 0:6 towards cerium ions from the uranium-containing solution. The prepared model solution was poured into 7 glasses (100 mL each). Then, the ion exchangers Amberlite IR120H and AV-17-8 were placed separately into the polypropylene mesh in accordance with the molar ratios X:Y (6:0, 5:1, 4:2, 3:3, 2:4, 1:5, and 0:6) to form an interpolymer system. For spectrometry analysis, one aliquot (1 mL) was taken from each glass at the set time. The aliquot sampling times were 0.1, 1, 2, 4, 6, 24, and 48 h. In total, 63 aliquots of solution were prepared for analysis.

For the inductively coupled plasma–optical emission spectrometry (ICP-OES) analysis, each aliquot (1 mL) with an unknown concentration of the analyte was transferred into the volumetric flasks (50 mL). Then, 1 mL of nitric acid (70%, purified by distillation) and 1 mL of standard cadmium solution were poured into each flask. Further, the volume of each solution was brought to 50 mL with distilled water, and after 15 min the measurements were started. It should be noted that the reference solution contained all of the above components with the exception of the analyte.

The concentrations of cerium ions in the solution, as well as the concentration of cerium ions sorbed by the ion exchangers, were determined using the ICP-OES technique. The sorption degree (η) was calculated using Equation (2):

(2) $$\eta =\frac{C_{\mathrm{initial}}-C_{\mathrm{residual}}}{C_{\mathrm{initial}}}\times 100\%$$

where C_initial_ and C_residual_ are the initial and residual concentrations (in g/L) of cerium ions in the solutions, respectively.

The desorption process was studied in the interpolymer system “Amberlite IR120:AV-17-8” (4:2), as this was the most effective system for cerium ion sorption among those studied. This process consisted of removing the sorbed cerium ions from the surface of the interpolymer system “Amberlite IR120:AV-17-8” (4:2) using 2M nitric acid (as eluent), which had a high affinity for the adsorbed REM [8] (p. 11).

The desorption degree (R) was calculated by the following Equation (3):

(3) $$R=\frac{m_{\mathrm{desorbed}}}{m_{\mathrm{sorbed}}}\times 100\%$$

## 3. Results

### 3.1. Determination of the Polymer Chain Binding Degree

The polymer chain binding degree (θ) indicates the ratio of the amount of cerium ions adsorbed to the total amount of ionic groups in the Amberlite IR120H and AV-17-8 ion exchanger interpolymer system. Table 1 shows the results calculated for the polymer chain binding degree of cerium ions by the interpolymer system Amberlite IR120:AV-17-8 (X:Y) at different pH values according to Equation (1). As can be seen from Table 1, the intensive binding of cerium ions by the interpolymer system Amberlite IR120:AV-17-8 (4:2) occurred at pH 2.0.

### 3.2. Electrochemical Studies of the Interpolymer System “Amberlite IR120:AV-17-8” (X:Y) in Aqueous Solutions

**Figure 2:**
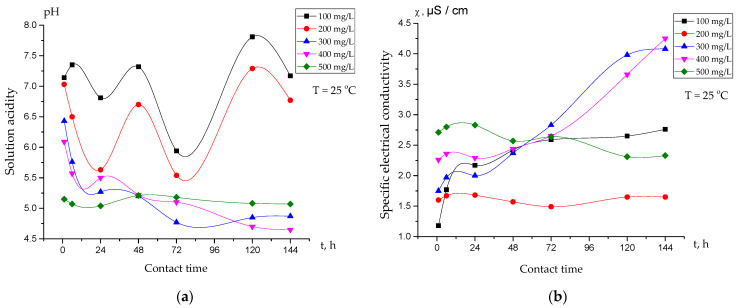
(**a**) Dependence of pH on the mass concentrations of Amberlite IR120 and AV-17-8 (3:3) in solutions as a function of time; (**b**) dependence of specific electrical conductivity (χ) on the mass concentrations of Amberlite IR120 and AV-17-8 (3:3) in solutions as a function of time.

Figure 2a demonstrates the dependence of the pH on the mass concentrations of the equimolar ratio of Amberlite IR120 and AV-17-8 (3:3) in the solutions as a function of time. Some of the decrease in the pH values during the first 24 h of interaction could be explained by the release of H^+^ ions into the solution as a result of the dissociation of functional sulfo-groups from the Amberlite IR120. After 24 h, fluctuations in the pH values, especially for interpolymer systems with polyelectrolyte mass concentrations of 100 mg/L and 200 mg/L, could be observed. The increase in pH values from 72 to 120 h could be explained by the binding of H^+^ ions from the solution by the nitrogen atom of the dissociated functional group of AV-17-8. The minor changes in the pH values after 120 h of interaction may have indicated the obtaining of an electrochemical equilibrium state in the solution.

Figure 2b shows the dependence of the specific electrical conductivity (χ) on the mass concentrations of Amberlite IR120 and AV-17-8 (3:3) in the solutions as a function of time. According to the obtained data, we observed a significant increase in the specific electrical conductivity (χ) values for interpolymer systems with polyelectrolyte mass concentrations of 300 mg/L and 400 mg/L, which could be explained by the appearance of new OH^−^ ions due to the expansion of additionally dissociated AV-17-8. The excess of OH^−^ ions resulted in increased values of specific electrical conductivity.

### 3.3. Influence of the pH on the Sorption Degree of Cerium Ions

Previous research [33] showed that the optimal time for the maximum degree of rare earth ion sorption by interpolymer systems was reached during the first 48 h. However, no study has been conducted on the effect of the pH on the sorption of cerium ions, in particular, from uranium-containing solutions.

**Figure 3:**
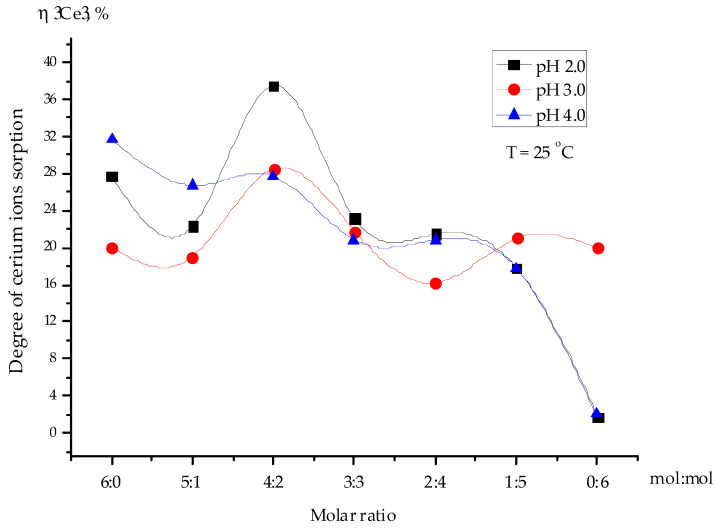
Dependence of cerium ion sorption extraction by the interpolymer system “Amberlite IR120:AV-17-8” (X:Y) (6:0, 5:1, 4:2, 3:3, 2:4, 1:5, and 0:6) on the pH of the solution as a function of the molar ratio X:Y after 48 h.

Figure 3 demonstrates the dependence of the cerium ion extraction degree by the interpolymer system “Amberlite IR120:AV-17-8” (X:Y) on the pH value as a function of the molar ratio. The data showed that the highest degree of cerium ion sorption (37%) by the interpolymer system “Amberlite IR120:AV-17-8” (4:2) occurred after 48 h of remote interaction at pH 2.0, whereas the raw Amberlite IR120 (6:0) and raw AV-17-8 (0:6) ion exchangers showed 27% and 2% cerium ion sorption, respectively, after 48 h of interaction. The obtained data suggested that the extraction of cerium ions occurred mainly due to the strongly acidic Amberlite IR120 H cation exchanger, as indicated by achievement of the highest ionization degree for this polyelectrolyte as a result of mutual activation.

Table 2 presents the results of several reports on the effect of the solution pH on the maximum capacity of different sorbents for cerium ions. According to Table 2, the majority of studies demonstrated that the most favorable pH range for maximum cerium sorption is 4–6. It is likely that the sorption properties of various types of sorbents are directly affected by the ionic form of cerium in aqueous solutions. In our case, the maximum sorption of the interpolymer system “Amberlite IR120:AV-17-8” (4:2) was observed at pH 2.0, with a maximum sorption capacity of the sorbent equal to 210.70 mg/g.

### 3.4. Selective Sorption of Cerium Ions from Uranium-Containing Solution by the Interpolymer System “Amberlite IR120:AV-17-8” (X:Y, Molar Ratio of Ionic Groups)

The study on the influence of the medium’s pH on the sorption of cerium ions revealed that the optimal conditions for the maximum sorption of the cerium ions occurred at pH 2.0 (Figure 3). For this reason, the sorption of cerium ions from the uranium-containing solution was implemented in a medium with a pH of 2.0.

**Figure 4:**
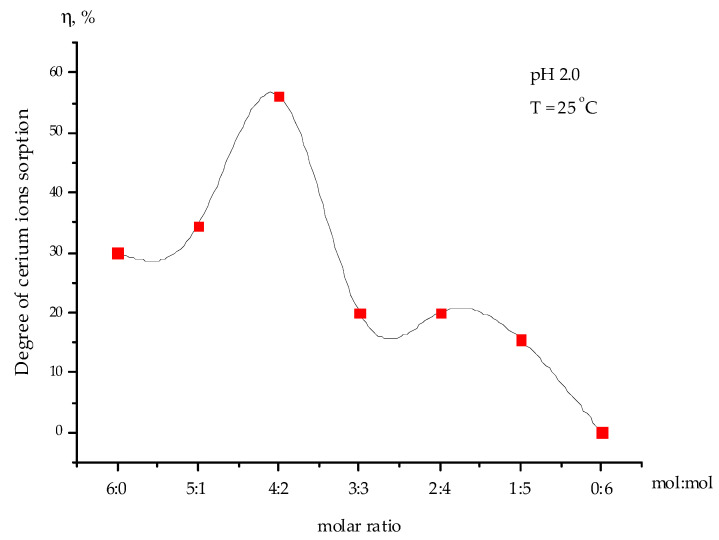
The sorption degree of cerium ions from the uranium-containing solution by the interpolymer system “Amberlite IR120H:AV-17-8” (X:Y) (6:0, 5:1, 4:2, 3:3, 2:4, 1:5, and 0:6) at pH 2.0 after 48 h of interaction.

Figure 4 shows the dependence of the sorption degree of cerium ions from the uranium-containing solution by the interpolymer system “Amberlite IR120 H:AV-17-8” (X:Y) at pH 2.0. As can be seen from this figure, with the combined presence of cerium and uranium ions in the solution, the highest cerium ion sorption ability was observed in the interpolymer system “Amberlite IR120:AV-17-8” at a molar ratio equal to 4:2. The maximum degree of cerium ion sorption after 48 h of remote interaction was 56%, while the cerium ion sorption degree by the raw Amberlite IR120 (6:0) and raw AV-17-8 were 30% and 0%, respectively.

To confirm the obtained data, an ICP-OES analysis of the residual concentration of cerium/uranium ions in the solutions and ion exchangers was additionally carried out. Table 3 presents the results of the ICP-OES analysis, which confirmed the increased cerium ion sorption activity of the interpolymer system “Amberlite IR120 H:AV-17-8” (4:2).

The ICP-OES analysis of the interpolymer system “Amberlite IR120H:AV-17-8” (4:2) (Table 3) confirmed the previously obtained data regarding cerium ion sorption (Figure 4). The experimental data confirmed the selective sorption of cerium ions from the uranium-containing solution by the interpolymer system “Amberlite IR120H:AV-17-8” (4:2).

The selective sorption of cerium ions by the interpolymer system “Amberlite IR120H:AV-17-8” (4:2) in the uranium-containing solution could be explained by Pearson’s hard-and-soft acids-and-bases (HSAB) concept [42], which states that “hard acids prefer to bond to hard bases, and soft acids prefer to bond to soft bases” [43]. In our case, in the cerium–uranium mixed ion solution, the interpolymer system “Amberlite IR120H:AV-17-8” (4:2) demonstrated selective affinity towards Ce^3+^ ions (hard acid), which are harder than ${\mathrm{UO}}_{2}^{2+}$ acids due to their higher ionization energy, and it was assumed that the hard base groups (RSO₂O⁻) of the ion exchanger played a significant role in the sorption process. We supposed that the sorption of uranyl ions (${\mathrm{UO}}_{2}^{2+}$) by the same interpolymer system was completely limited due to the ion size and the sorption site limitations of the adsorbent for bulky and softer acid uranyl ions.

### 3.5. The Kinetics of Cerium Ion Desorption from the Interpolymer System “Amberlite IR120:AV-17-8” (4:2)

**Figure 5:**
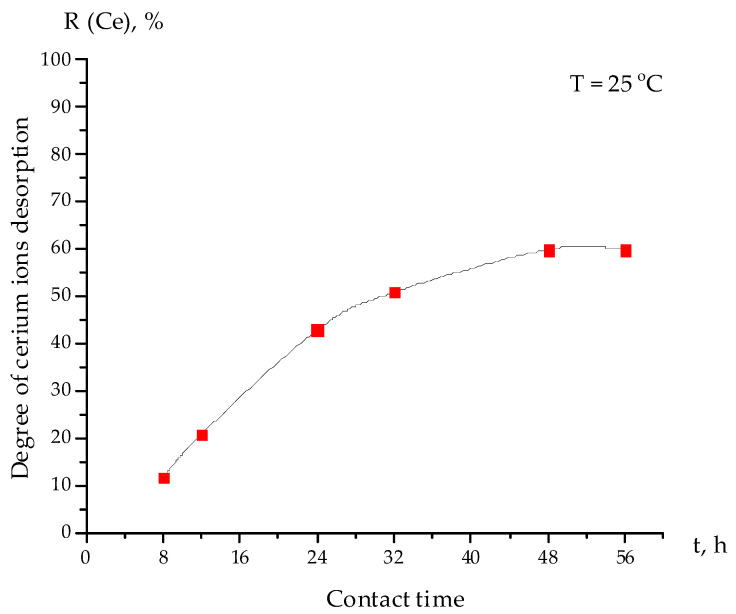
Kinetics of cerium ion desorption from the interpolymer system “Amberlite IR120H:AV-17-8” (4:2).

To study the process of cerium ion desorption from the interpolymer system “Amberlite IR120H:AV-17-8” (4:2), 2M nitric acid was prepared and used as a desorbing agent (eluent). The kinetics of the cerium ion desorption are shown in Figure 5. The desorption degree of cerium ions R (Ce) (%) was calculated according to Equation (3). As can be seen from Figure 5, the highest value of cerium ion desorption (60%) occurred after 48 h of the desorption process.

## 4. Conclusions

In this research, the selective recovery of cerium ions from a uranium-containing solution by the remotely activated ion exchangers Amberlite IR120H and AV-17-8 (forming the interpolymer system “Amberlite IR120H:AV-17-8” (X:Y) (6:0, 5:1, 4:2, 3:3, 2:4, 1:5, and 0:6)) was investigated. The presented study revealed the influence of the pH of the solution on the sorption activity of the interpolymer system. The optimal conditions for cerium ion sorption were observed at pH 2.00. After 48 h of interaction, the highest degree of cerium ion sorption by the interpolymer system “Amberlite IR120H:AV-17-8” (4:2) was 56%, whereas the cerium ion sorption by raw Amberlite IR120H (6:0) and raw AV-17-8 (0:6) was 30% and 0%, respectively. The maximum sorption capacity of the interpolymer system “Amberlite IR120H:AV-17-8” (4:2) was equal to 210.70 mg/g. Moreover, the maximum cerium ion desorption degree using 2M nitric acid as a desorbing agent (eluent) after 48 h was 60%. However, the increased cerium ion sorption by the abovementioned interpolymer system in comparison with the raw (unactivated) polyelectrolytes could be explained by the achievement of a high degree of ionization in this system, resulting from remote interaction in the aqueous medium. The obtained results demonstrated the potential use of the interpolymer system “Amberlite IR120H:AV-17-8” (4:2) as a promising polymer sorbent for cerium ion sorption from solutions containing radioactive elements as contaminants.

The main improvements made by this research compared to the findings in the literature lie in the application of the remote activation effect, which contributes to the development of a set of conformational states in polymers with a certain distribution of complementary structures to various REM ion radii.

For future work and to better assess cerium ion recovery from secondary sources and the feasibility of the sorption process on an industrial scale, the following topics should be addressed: (i) the economic assessment of the sorption/desorption processes and their mechanisms, (ii) the regeneration of interpolymer systems, and (iii) further studies on their applications.

## Figures and Tables

**Table 1:** The polymer chain binding degree (θ, %) of cerium ions by the interpolymer system Amberlite IR120:AV-17-8 according to the molar and weight ratios (X:Y) and pH values of the solutions (T = 25 °C).

Molar Ratio of Amberlite IR120:AV-17-8 (X:Y)	Weight Ratio of Amberlite IR120:AV-17-8 (X:Y) ^a^	θ at pH 2.0	θ at pH 3.0	θ at pH 4.0
6:0	0.207 g:0.000 g	5.93%	4.28%	6.78%
5:1	0.172 g:0.024 g	4.77%	4.06%	5.71%
4:2	0.138 g:0.049 g	8.02%	6.10%	5.93%
3:3	0.103 g:0.073 g	4.96%	4.66%	4.45%
2:4	0.069 g:0.098 g	4.60%	3.47%	4.45%
1:5	0.034 g:0.122 g	3.81%	4.49%	3.81%
0:6	0.000 g:0.146 g	0.36%	4.28%	0.43%

Conditions: initial concentration of Ce = 6.03 mg/L each, 25 °C, 48 h. ^a^ Ratio of weight of components of interpolymer system determined by the gravimetry method.

**Table 2:** The effect of the solution pH on the maximum capacity of various sorbents for cerium ions.

Sorbents	PH Range	Optimum PH	Maximum Sorption Capacity (mg/g)	Reference
Poly(allylamine)/silica composite	1.0–6.0	4.0	111.80	[34]
Rice husk grafted poly (methyl acrylic acid)	1.0–7.0	6.0	122.51	[35]
Magnetic nanocomposite hydrogel	1.0–4.0	4.0	151.00	[36]
ZrT hybrid ion exchanger	1.0–5.0	5.0	112.00	[37]
*Bacillus licheniformis*	1.0–6.0	6.0	38.93	[38]
Biopolymeric-layered double hydroxides hybrid nanocomposites	2.0–7.0	4.0	116.82	[39]
Hydrous zirconium oxide	0.0–1.0	~0.5	184.50	[40]
Ligand immobilized nano-composite	1.0–7.0	2.5	150.37	[41]
Interpolymer system “Amberlite IR120:AV-17-8” (4:2)	2.0–4.0	2.0	210.70	Current study

**Table 3:** Amount of Ce and U ions adsorbed by the interpolymer system “Amberlite IR120H:AV-17-8” (4:2) under different pH values.

Ion	Amount of Ions (mg/g) ^a^		
	pH = 2.0	pH = 3.0	pH = 4.0
Ce	1.293	1.138	1.112
U	0.001>	0.001>	0.001>

Conditions: initial concentration of Ce and U = 6.03 mg/L each, adsorbent 187.00 mg/L, 25 °C, 48 h. ^a^ Ratio of weight of ions adsorbed to weight of adsorbent determined by ICP-OES.
